# Polo-Like Kinase 2 Plays an Essential Role in Cytoprotection against MG132-Induced Proteasome Inhibition via Phosphorylation of Serine 19 in HSPB5

**DOI:** 10.3390/ijms231911257

**Published:** 2022-09-24

**Authors:** Shuji Ueda, Moeka Nishihara, Yuuki Hioka, Ken-ichi Yoshino, Soichiro Yamada, Minoru Yamanoue, Yasuhito Shirai

**Affiliations:** 1 Department of Agrobioscience, Graduate School of Agricultural Science, Kobe University, Kobe 657-8501, Japan; 2 Biosignal Research Center, Kobe University, Kobe 657-8501, Japan; 3 Department of Biomedical Engineering, University of California Davis, Davis, CA 95616, USA

**Keywords:** heat shock protein, PLK2, ER stress, PARP, desmin-related cardiomyopathy, MG132, BioID2, tamavidin

## Abstract

Protein homeostasis, including protein folding, refolding, and degradation, is thought to decline with aging. HSPB5 (also known as αB-crystallin) prevents target protein aggregation as a molecular chaperone and exhibits a cytoprotective function against various cell stresses. To elucidate the effect of HSPB5 on endoplasmic reticulum (ER) stress, we searched for novel binding proteins of HSPB5 using the proximity-dependent biotin labeling method. Proteins presumed to interact with HSPB5 in cells treated with the proteasome inhibitor MG132 were identified by a reversible biotin-binding capacity method combining tamavidin2-REV magnetic beads and mass spectrometry. We discovered a new binding protein for HSPB5, polo-like kinase 2 (PLK2), which is an apoptosis-related enzyme. The expression of PLK2 was upregulated by MG132 treatment, and it was co-localized with HSPB5 near the ER in L6 muscle cells. Inhibition of PLK2 decreased ER stress-induced phosphorylation of serine 19 in HSPB5 and increased apoptosis by activation of caspase 3 under ER stress. Overexpression of HSPB5 (WT) suppressed the ER stress-induced caspase 3 activity, but this was not observed with phospho-deficient HSPB5 (3A) mutants. These results clarify the role of HSPB5 phosphorylation during ER stress and suggest that the PLK2/HSPB5 pathway plays an essential role in cytoprotection against proteasome inhibition-induced ER stress.

## 1. Introduction

The endoplasmic reticulum (ER) is a network of membranous structures extending from the nuclear membrane to the cytosol, and is responsible for homeostasis in protein synthesis, transport, and folding. ER stress is caused by the accumulation of denatured proteins owing to various factors. The ER is equipped with three transmembrane proteins (PERK, IRE1, and ATF6), which transmit the unfolded protein response (UPR) to the nucleus. UPR signaling restores protein homeostasis by regulating the rate of protein synthesis and enhancing the transcription of ER chaperone proteins and their regulatory proteins, whereas the activation of ER-associated degradation degrades denatured proteins in the proteasome system. However, the accumulation of severe or chronic ER stress cannot be recovered by UPR, and it is known to cause cell apoptosis as a self-protective response.

It has been reported that proteasome dysfunction induces ER stress through decreased degradation of denatured proteins. Diabetes, neurodegenerative diseases, metabolic diseases, cancer, and hypertension are closely related to ER stress associated with aging and genetic factors [1]. Recently, in skeletal muscle tissue, increased ER stress has been reported in myopathies, such as muscular dystrophy and inclusion body inflammation [2]. Chronic ER stress owing to aging is also considered a potential factor in sarcopenia, an age-related muscular atrophy [3]. In the tissues of these diseases, cell apoptosis is induced via transcription of C/EBP homologous protein (CHOP) as a UPR against the accumulation of denatured proteins [4]. Thus, proteasome dysfunction with aging has been suggested to be closely related to the development and prognosis of ER stress-related diseases [5].

The small heat shock protein (sHSP) family is known to have “chaperone activity” with some sHSPs preventing the aggregation of denatured proteins. sHSPs have a highly conserved α-crystallin domain, and ten isoforms (HSPB1 to HSPB10) are known in mammals. sHSP formation is induced by various intracellular stresses and regulates protein degradation and folding. HSPB5 (also known as αβ-crystallin) is expressed predominantly in the lens of the eye, cardiac muscle, and skeletal muscle [6]. HSPB5-knockout mice exhibit severe progressive myopathy with aging [7], and a point mutation, HSPB5 (R120G), has also been reported to cause desmin-related myopathy in humans [8]. HSPB5, together with HSPB1 (also known as HSP27), forms oligomers [9] and acts as a chaperone, and it is thought to play an essential role in maintaining muscle tissue homeostasis. HSPB5 has three phosphorylation sites (serine 19, serine 45, and serine 59). Serine 59 of HSPB5 is phosphorylated by MAP kinase-activated protein kinase (MK2), and serine 45 is thought to be phosphorylated by p38 MAP kinase, but the kinase phosphorylating serine 19 (pSer19) is still unknown [10,11]. These phosphorylation-deficient mutants impair the oligomerization of HSPB5 and HSPB1, suggesting that these phosphorylation sites increase the affinity of HSPB5 for denatured proteins [12]. However, the phosphorylation role of HSPB5 in ER stress remains unknown.

BioID is a proximity biotinylation labeling method using the bacteria-derived BirA enzyme and enabling the comprehensive identification of protein interactions in cells [13,14]. This BioID method has the advantage that biotin modification can be performed in living cells by expressing the bait protein as a BirA fusion protein, and thus, various biological reactions can be observed in real-time [15,16]. In this study, to explore the regulatory mechanism of the molecular chaperone HSPB5 in preventing the unfolding and aggregation of target proteins under ER stress, we comprehensively analyzed the proteins that interact with HSPB5 in cells after treatment with the proteasome inhibitor MG132, which induces ER stress [17], using the BioID method. As a result, proteins related to the ubiquitin-proteasome system, neurodegenerative diseases, and cytoskeleton were identified as candidate proteins interacting with HSPB5 under ER stress conditions. In this study, we report that polo-like kinase 2 (PLK2), the first kinase that phosphorylates serine 19 on HSPB5, inhibits ER stress-induced cell apoptosis via pSer19 of HSPB5.

## 2. Results

### 2.1. Search for HSPB5 Binding Proteins by Proximity-Dependent Biotin Labeling

**Figure 1:**
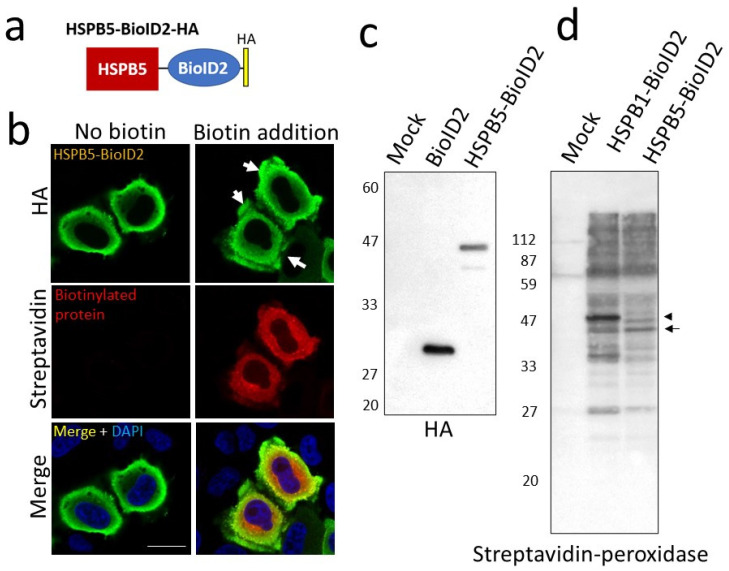
Construction of HSPB5-BioID2 expression plasmid and BioID activity. (**a**) Schematic diagram of BioID2 fusion protein. (**b**) Subcellular localization of HSPB5-BioID2 expression. HeLa cells were transfected with expression plasmid, and 24 h after addition of biotin (50 μM), cells were immunostained with anti-HA antibody and stained with fluorescently labeled secondary antibody, streptavidin-Alexa Fluor 546, and with 4’,6-diamidino-2-phenylindole dihydrochloride (DAPI). White arrows indicate lamellipodia around cell membrane. Scale bar indicates 20 μm. (**c**) Detection of BioID2 fusion protein expression and BioID activity. 293T cells were transfected with expression plasmid, and cells were collected 24 h after addition of biotin. Expression of BioID2 fusion protein was detected with an anti-HA antibody. (**d**) Biotinylated proteins were detected using streptavidin−peroxidase polymer. Arrowheads (HSPB1-BioID2) and arrows (HSPB5-BioID2) indicate positions of respective proteins. HSPB1 is an isoform that belongs to the same sHsp family as HSPB5.

As protein homeostasis decreases with aging, ER stress increases, owing to the accumulation of denatured proteins in cells. sHSP expression has been reported to increase with age in the skeletal muscle [18,19]. There is limited knowledge of the cytoprotection of HSPB5 in ER stress. In this study, to elucidate the role of HSPB5 in ER stress caused by proteasome inhibition, we explored the binding proteins involved in the chaperone function of HSPB5 under ER stress. Protein–protein binding was detected by enzymatic labeling using promiscuous biotin ligase derived from *Aquifex aeolicus* (BioID2) [20], which is substantially smaller and more efficient at biotin labeling than the conventional BirA from *Escherichia coli* [21]. The recombinant HSPB5 protein with BioID2 fused to its C-terminal side (HSPB5-BioID2) localized to the cytoplasm and lamellipodia, similar to endogenous HSPB5. HSPB5-BioID2 co-localized with the biotinylated protein after 24 h incubation with biotin (Figure 1a,b). In addition, Western blotting increased biotinylated proteins of various molecular weights by expressing HSPB5-BioID2 and HSPB1-BioID2 (Figure 1c,d).

**Figure 2:**
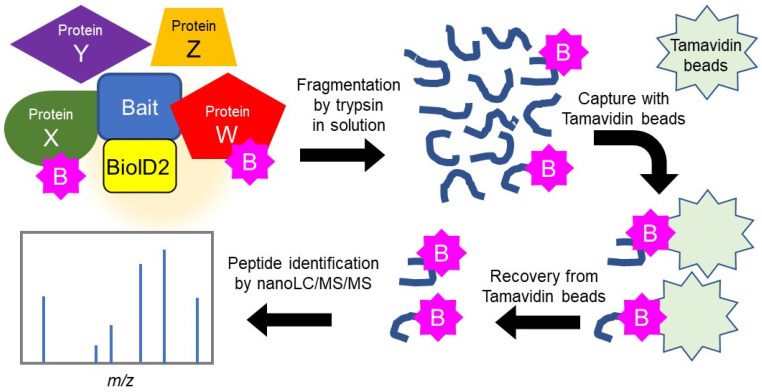
Efficient protein–protein binding analysis using BioID2 and Tamabidine 2-REV beads. Schematic diagram of a method for identifying biotinylated proteins in a proximity-dependent manner by BioID2 fusion proteins.

To improve the efficiency of protein identification, we purified biotinylated proteins after degradation by trypsin protease by following the “direct detection of biotin-containing tags” (DiDBit) method [22] to reduce contamination by non-specific pseudo-binding proteins. We also employed tamavidin 2-REV magnetic beads (dissociation constant 6 × 10^−7^ M), which have a lower affinity for biotin than streptavidin (6 × 10^−16^ M), and eluted the bound biotinylated peptides from the beads under mild conditions [23]. These innovations facilitated the comprehensive analysis of the proteins bound to HSPB5 by nanoscale liquid chromatography coupled to tandem mass spectrometry (nanoLC/MS/MS) (Figure 2). To detect binding proteins, we transiently overexpressed HSPB5-BioID2 in 293T cells and compared the biotinylated proteins with and without ER stress by treatment with MG132 (5 μM) for 24 h. Among the peptides reproducibly detected by nanoLC/MS/MS analysis, the proteins whose exponentially modified protein abundance index (emPAI) scores were increased by MG132 treatment are shown (Table 1). By the proximity-dependent biotin labeling method, we detected 16 new candidate proteins that bind to HSPB5.

### 2.2. Validation of a New Binding Protein, PLK2

**Figure 3:**
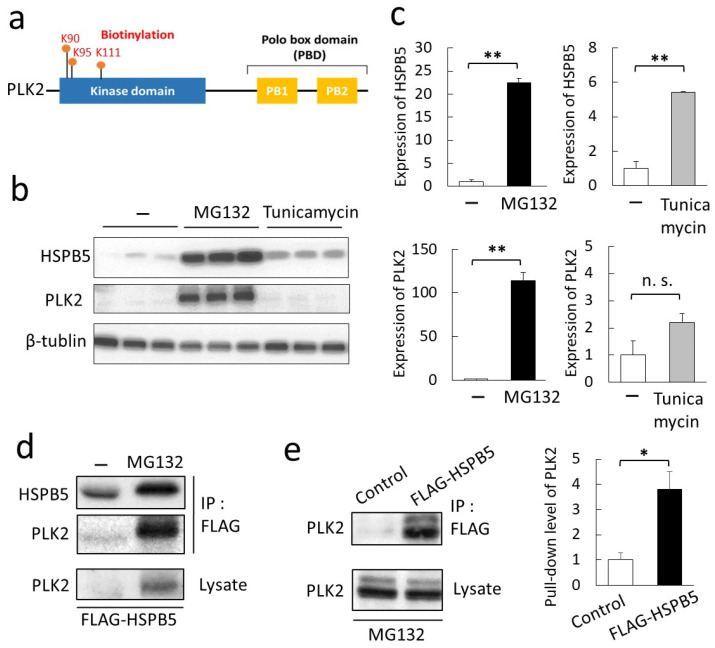
PLK2, a novel binding protein of HSPB5, is induced by MG132 in L6 cells. (**a**) Schematic diagram of domain structure of PLK2. K90, K95, and K111 indicate position of lysine residue (K) biotinylated by HSPB5-BioID2. Lysine residues were numbered based on amino acid sequence of PLK2 derived from *Homo sapiens* (NCBI NP_006613). (**b**) Comparison of induction of HSPB5 and PLK2 expression by ER stress-induced drugs. Differentiated L6 cells were treated with 5 µM MG132 or 0.1 µg/mL tunicamycin for 24 h. Induction of HSPB5, and PLK2 protein expression was detected by Western blotting. (**c**) Graph shows levels of endogenous proteins induced by the drug. Data represent means ± standard error (n = 3). Statistical analyses were performed using *t*-test. ** *p <* 0.01; n.s. means not significant. (**d**) Verification of binding of PLK2 to HSPB5. FLAG-HSPB5 was overexpressed in L6 cells by liposome transfection. After 5 µM MG132 treatment (24 h), cells were solubilized, and HSPB5 pull-down assay was performed using an anti-tag antibody. (**e**) Quantification of binding of PLK2 to HSPB5. Control cells were transfected with empty vector. After 5 µM MG132 treatment (24 h), a pull-down assay was performed using an anti-tag antibody. Graph presents level of endogenous PLK2 pulled down by HSPB5. Data represent means ± standard error (n = 3). Statistical analyses were performed using *t*-test. * *p <* 0.05.

We focused on PLK2 among the candidate proteins predicted to bind HSPB5 by MG132 treatment. PLK2 belongs to the “Polo” family of serine/threonine kinases and functions in mitosis and cell cycle regulation [24]. PLK2 showed a significant value of emPAI, as acquired by mass spectrometry, and emPAI increased with ER stress (Table 1). Mass spectrometry analysis confirmed that the 90th, 95th, and 111th lysine residues of PLK2, located on the N-terminal side of the kinase domain, were biotinylated (Figure 3a). Analysis of the biotinylated peptide of PLK2 by mass spectrometry is presented (Figure S1). The protein expression of PLK2 was synchronized with the increased expression of HSPB5 following MG132 treatment (Figure S2). Tunicamycin is a drug used to induce ER stress by inhibiting glycosyltransferases [17]. We compared the induction of HSPB5 and PLK2 expression with MG132 and tunicamycin. MG132 treatment upregulated the expression of HSPB5 and PLK2, while the tunicamycin treatment upregulated HSPB5 expression as well as MG132, but showed no significant difference in PLK2 expression (Figure 3b,c). Upregulation of PLK2 was also confirmed by bortezomib [25], another proteasome inhibitor, as well as MG132 treatment (Figure S3). These results indicate that the upregulation of PLK2 is specific to proteasome inhibition by MG132 treatment.

Since PLK2 expression is upregulated in an MG132 treatment-dependent manner, verification of the binding of HSPB5 to PLK2 was enabled only in a pull-down assay using MG132-treated cells. Endogenous PLK2 was upregulated in L6 cells after MG132 treatment, and the PLK2 was verified to bind directly or non-directly to FLAG-HSPB5 (Figure 3d,e).

### 2.3. Phosphorylation of HSPB5 by PLK2 Triggered by ER Stress via Proteasome Inhibition

**Figure 4:**
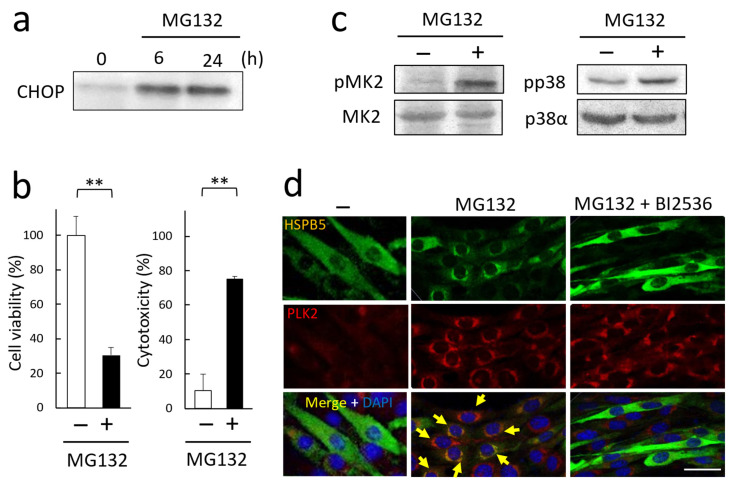
MG132 induced ER stress in L6 cells. (**a**) Induction of ER stress by MG132. After differentiation (48 h), L6 myotubes were treated with 5 µM MG132. Induction of CHOP expression was detected by Western blotting. (**b**) Effect of MG132 treatment on cell viability and cytotoxicity of L6 cells. After differentiation, L6 myotubes were treated with 5 µM MG132 (24 h). Cytotoxicity was measured from activity of lactate dehydrogenase in the medium. Data in graphs represent means ± standard error (n = 3). Statistical analyses were performed using *t*-test. ** *p <* 0.01. (**c**) Activation of stress-related kinases by MG132 treatment. After 5 µM MG132 treatment (24 h), phosphorylation of MK2 (pThe334) and p38 (pThr180/pTyr182) was detected by Western blotting. (**d**) Colocalization of endogenous HSPB5 and PLK2. After differentiation (48 h), endogenous HSPB5 and PLK2 were immunofluorescently stained and observed by confocal microscopy. BI2536 is a PLK-specific inhibitor. BI2536 (10 nM) was treated simultaneously with 5 µM MG132 for 24 h. Yellow arrows indicate cells with co-localization of HSPB5 and PLK2 in perinuclear ER region. Scale bar indicates 20 μm.

Further, we examined the conditions of ER stress in L6 myotubes after myogenic differentiation. After MG132 treatment, the induction of CHOP, an ER stress marker, was detected at 6 h (Figure 4a), and L6 cells decreased cell viability and increased cytotoxicity after 24 h (Figure 4b). MG132 treatment also promoted the activation of stress-related kinases such as MK2 and p38 MAP kinases (p38) in L6 myotubes (Figure 4c). The subcellular localization of HSPB5 and PLK2 was observed by confocal microscopy in differentiated L6 cells. In myotubes, HSPB5 expression was upregulated upon differentiation [26], and HSPB5 localized throughout the cytoplasm. In contrast, PLK2 expression was not upregulated by differentiation. After MG132 treatment, PLK2 expression was upregulated until it was detected by confocal microscopy, and HSPB5 and PLK2 co-localized in the ER periphery around the perinucleus (Figure 4d). HSPB5 is thought to co-localize in the ER region with the ER marker, protein disulfide isomerase (PDI) (Figure S4). This colocalization of HSPB5 and PLK2 was suppressed by BI2536 treatment, a specific kinase-activity inhibitor of PLKs [27]. BI2536 specifically inhibited the transfer of HSPB5 from the cytoplasm to the ER region by MG132 treatment. In contrast, BI2536 did not affect the localization of PLK2-induced expression by MG132 treatment. This suppression suggests that phosphorylation of some proteins by PLK2 kinase activity is involved in the translocation to the ER region of HSPB5 under ER stress.

**Figure 5:**
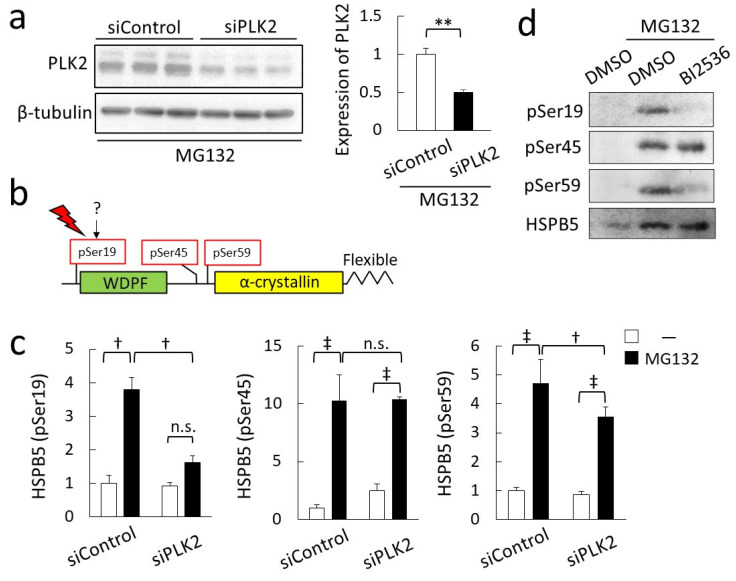
PLK2 phosphorylates HSPB5 at serine 19 under ER stress. (**a**) Knockdown efficiency of PLK2 by siRNA. L6 cells were transfected with siRNA to knockdown PLK2 and treated with 5 µM MG132 for 24 h. Graph presents relative value of expression level of PLK2. Data in graphs represent means ± standard error (n = 3). Statistical analyses were performed using *t*-test. ** *p <* 0.01. (**b**) Schematic representation of three known phosphorylation sites of HSPB5. Arrows indicate name of the kinase catalyzing phosphorylation. (**c**) Effect of PLK2 on each phosphorylation site. Undifferentiated L6 cells were transfected with siRNA to knockdown PLK2. After differentiation (48 h), L6 myotubes were treated with 5 µM MG132 for 24 h. Phosphorylation level was detected by Western blotting with specific antibodies. Graph presents ratio of phosphorylated HSPB5/HSPB5 after quantification of each band. Data in graphs represent means ± standard error (n = 3). Statistical analyses were performed using one-way ANOVA with Tukey test. ‡ *p <* 0.01, † *p <* 0.05; n.s. not significant. (**d**) Effect of PLK2 activity on phosphorylation of serine 19. After differentiation (48 h), L6 myotubes were treated with 10 nM BI2536 and 5 µM MG132 for 24 h. Phosphorylation level was detected by Western blotting with specific antibodies.

We examined the phosphorylation of HSPB5 by PLK2 using small interfering RNA (siRNA) knockdown. As MG132 treatment strongly induced PLK2 expression, the knockdown efficiency of PLK2 was only about 50%, but it was sufficient to study the ER stress response (Figure 5a). HSPB5 has three phosphorylation sites (Figure 5b), and it has been reported that these phosphorylation sites are involved in the chaperone function of HSPB5 [22]. In differentiated L6 myotubes, HSPB5 was significantly phosphorylated at three sites—serine 19, serine 45, and serine 59—by MG132 treatment. Knockdown of PLK2 suppressed the phosphorylation of HSPB5 at serine 19 and serine 59 following MG132 treatment. A comparison of serine 19 and serine 59 showed that PLK2 knockdown significantly suppressed phosphorylation at serine 19 (Figure 5c). This phosphorylation of serine 19 was also confirmed by inhibiting PLK activity by BI2536 treatment (Figure 5d).

### 2.4. Critical Role of HSPB5 and PLK2 in ER Stress Response via Proteasome Inhibition

**Figure 6:**
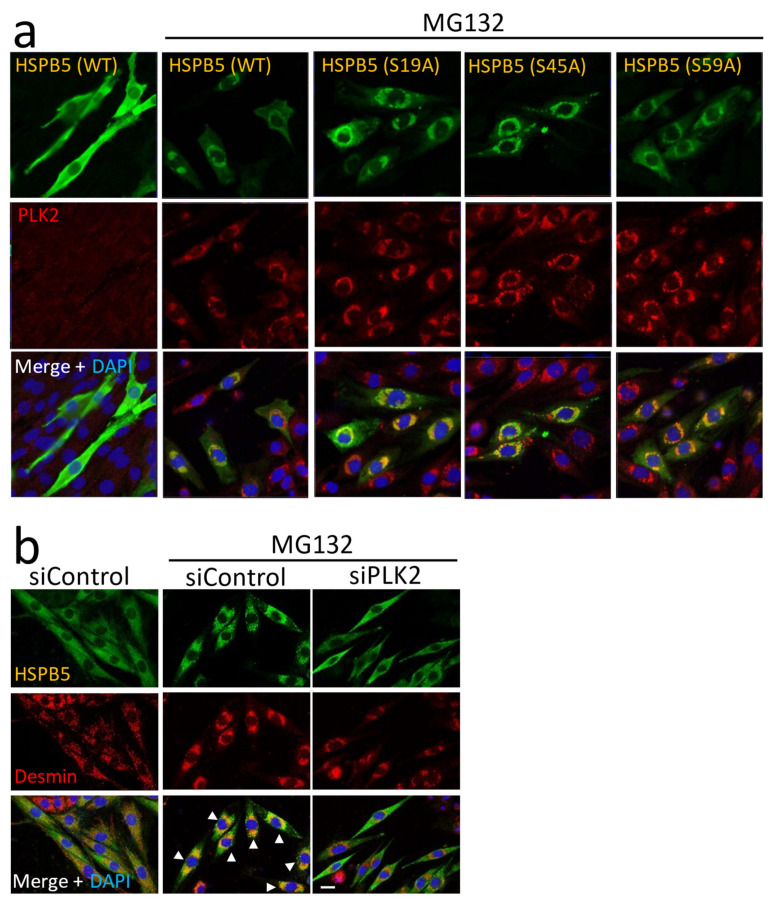
Relationship between phosphorylation and subcellular localization of HSPB5. (**a**) Localization of overexpressed recombinant HSPB5 and endogenous PLK2. After 5 µM MG132 treatment (24 h), FLAG-tagged HSPB5 and endogenous PLK2 in undifferentiated L6 cells were immunofluorescently stained with anti-tag and anti-PLK2 antibodies. (**b**) Effect of PLK2 on localization of HSPB5 and desmin protein. Endogenous HSPB5 and desmin were immunofluorescently stained with anti-HSPB5 and anti-desmin antibodies. Undifferentiated L6 cells were transfected with siRNA to knockdown PLK2. After differentiation (48 h), L6 myotubes were treated with MG132 for 24 h. White arrowheads indicate cells with colocalization of HSPB5 and desmin at perinuclear ER region. Scale bar indicates 20 μm.

Further, we examined the role of HSPB5 phosphorylation using a single amino acid mutant. Confocal microscopy images presented little difference in subcellular localization among overexpressed HSPB5 (WT), HSPB5 (S19A), HSPB5 (S45A), and HSPB5 (S59A). Namely, the HSPB5 mutants were localized in the ER region around the perinucleus, regardless of the amino acid substitution (Figure 6a). The localization pattern of HSPB5 in the ER region was similar to that of the HSPB5 (R120G) mutant, which causes familial desmin-related myopathies. Since the HSPB5 (R120G) mutant is known to abnormally localize with intracellular aggregations and intermediate filament desmin [28], we examined the localization of desmin and HSPB5 during ER stress. Desmin is an intermediate filament abundantly expressed in muscle [29] and observed in fibrous structures in the cytoplasm in L6 myotubes [30]. ER stress by MG132 treatment caused desmin to aggregate around the ER and HSPB5 colocalized with the aggregated desmin (Figure 6b). This colocalization of HSPB5 and aggregated desmin in the ER region inhibited the siRNA-mediated knockdown of PLK2. This subcellular localization suggests that phosphorylation of HSPB5 by PLK2 is essential for repairing denatured proteins around the ER (Figure 4d and Figure 6b).

**Figure 7:**
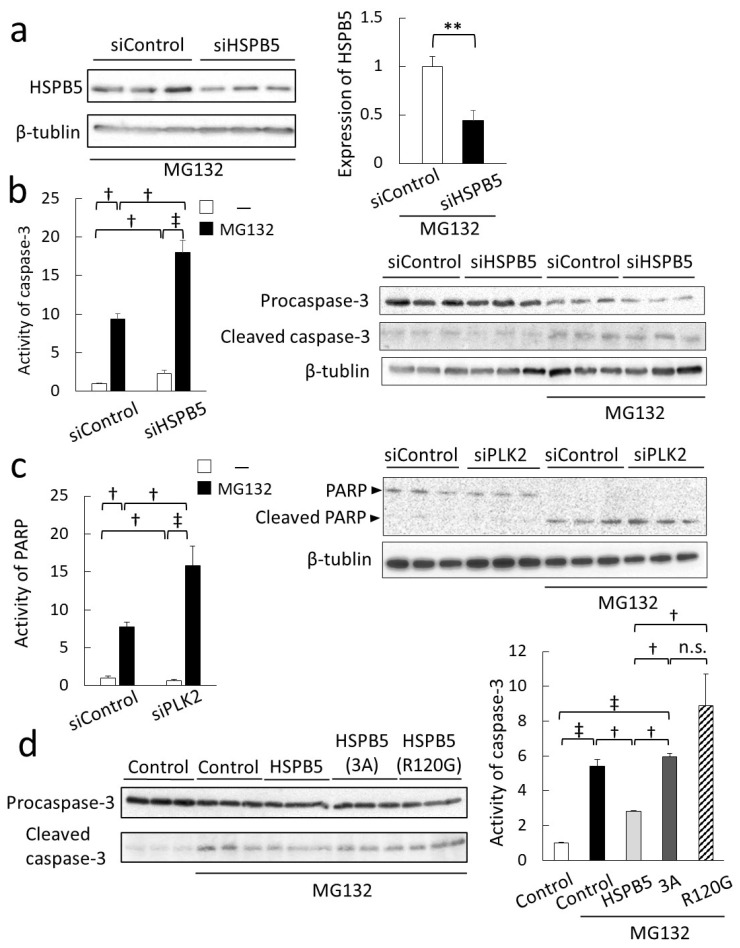
HSPB5 is involved in inhibition of caspase 3 via phosphorylation. (**a**) Knockdown efficiency of HSPB5 by siRNA. L6 cells were transfected with siRNA to knockdown HSPB5 and treated with 5 µM MG132 for 24 h. Graph presents relative value of expression level of HSPB5. (**b**) Effect of HSPB5 on ER stress-induced caspase 3 activity. Undifferentiated L6 cells were transfected with siRNA by lipofection. After differentiation (48 h), L6 myotubes were treated with MG132 for 24 h. Undifferentiated L6 cells were transfected with siRNA to knockdown HSPB5. After differentiation, L6 myotubes were treated with 5 µM MG132 for 24 h. Caspase 3 activity was quantified from ratio of procaspase 3 to cleaved caspase 3 (cleaved caspase 3/procaspase 3) by Western blotting using specific antibodies. Graph shows ratio of cleaved caspase 3/procaspase 3 after quantifying each band, relative toMG132 untreated siRNA control. (**c**) Effect of PLK2 on ER stress-induced PARP activity. Undifferentiated L6 cells were transfected with siRNA by lipofection. After differentiation (48 h), L6 myotubes were treated with 5 µM MG132 for 24 h. Undifferentiated L6 cells were transfected with siRNA to knockdown HSPB5. After differentiation (48 h), L6 myotubes were treated with MG132 for 24 h. PARP activity was quantified from ratio of PARP to cleaved PARP by Western blotting. Graph shows cleaved PARP/PARP ratio after quantifying each band relative to MG132 untreated siRNA control. (**d**) Inhibitory effect of HSPB5 protein on ER stress-induced caspase 3 activity. Undifferentiated L6 cells were transfected with expression plasmid by lipofection. Control cells were transfected with empty expression vector. After differentiation, L6 myotubes were treated with 5 µM MG132 for 24 h. Graph shows ratio of cleaved caspase 3/procaspase 3 after quantifying each band, relative to MG132 untreated control. Data in graphs represent mean ± standard error (n = 3). Statistical analyses were performed using one-way ANOVA with Tukey test or *t*-test. ** *p <* 0.01 (*t*-test). ‡ *p <* 0.01, † *p <* 0.05; n.s. means not significant (Tukey test).

Next, we examined the protective role of HSPB5 phosphorylation against ER stress-induced apoptosis by MG132 treatment. First, the effect of knockdown of HSPB5 by siRNA was evaluated by its effect on apoptosis. MG132 treatment induced the expression of HSPB5 and PLK2; therefore, the knockdown efficiency of HSPB5 remained at approximately 60%, but it did not interfere with this experiment (Figure 7a). ER stress-induced apoptosis via proteasome inhibition is caused by the coordination of the CHOP cascade and the caspase cascade, as well as other ER stresses [31]. Therefore, we evaluated the ratio of procaspase 3 to cleaved caspase 3 as an indicator of apoptosis of ER stress induced by MG132 treatment [32]. As expected, cleaved caspase 3 levels were increased by MG132 treatment in differentiated L6 myotubes (Figure 7b). In comparison with control siRNA, knockdown of HSPB5 significantly induced caspase 3 activity (siControl [–] vs. siHSPB5 [−]; † *p* < 0.05) and markedly increased caspase 3 activity (siHSPB5 [−] vs. siHSPB5 [MG132]; ‡ *p <* 0.01) after MG132 treatment (Figure 7b). Next, we examined the effect of PLK2 on ER stress-induced apoptosis. The effect of siRNA-mediated knockdown of PLK2 on the activation of poly ADP-ribose polymerase (PARP) [33], the most downstream caspase cascade [34], was evaluated (Figure 7c). Compared to control siRNA, knockdown of PLK2 significantly induced PARP activity (siControl [−] vs. siPLK2 [−]; † *p* < 0.05), and similar to the results of HSPB5 knockdown, PARP activity was significantly increased after MG132 treatment (siPLK2 [−] vs. siPLK2 [MG132]; ‡ *p* < 0.01).

Since HSPB5 has been suggested to function as a chaperone for denatured proteins under ER stress (Figure 6b) [35,36], we further examined the inhibitory effect of recombinant HSPB5 on ER stress-induced caspase 3 activity. Overexpression of HSPB5 (WT) significantly suppressed ER stress-induced caspase 3 activity after MG132 treatment (Figure 7d). In contrast, the expression of triple mutant HSPB5 (3A), unlike HSPB5 (WT), did not suppress caspase 3 activity (HSPB5 (WT) vs. HSPB5 (3A); † *p <* 0.05). Because HSPB5 (R120G) mutations in desmin-related myopathies are known to induce apoptosis by promoting protein aggregation [37], overexpression of HSPB5 (R120G) also confirmed the loss of the suppressive effect on ER stress-induced caspase 3 activation (HSPB5 (WT) vs. HSPB5 (R120G); † *p <* 0.05), as well as the HSPB5 (3A) triple mutation. In summary, these results suggest that phosphorylation of HSPB5 by PLK2 is essential for the repair of aggregated proteins around the ER and functions to suppress ER stress-induced apoptosis via proteasome inhibition.

## 3. Discussion

ER stress in skeletal muscle increases with aging. Chronic aging-associated ER stress causes muscle diseases, such as myopathy, by promoting apoptosis. The proteasome system is essential for protein homeostasis, selectively degrading denatured proteins. Aging-induced proteasome dysfunction is closely associated with protein-denaturing diseases in the elderly [38]. Therefore, it is crucial to understand the mechanism of cell protection against ER stress via proteasome inhibition at the molecular level [3]. The sHSP family of proteins is abundantly expressed in skeletal muscles and appears to be involved in protein remodeling in myofibers [26]. Age-related damage to cytoskeletal filaments increases the requirement for chaperone activity, and expression-induced HSPB5 may prevent the accumulation of harmful protein aggregates [39].

**Figure 8:**
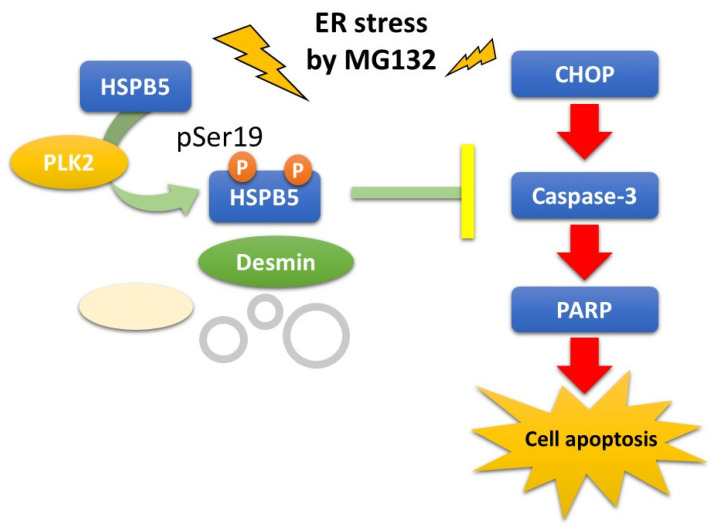
A novel PLK2/HSPB5 pathway is proposed as a model for cytoprotection against ER stress by MG132. PLK2, a novel binding protein of HSPB5, phosphorylates serine 19 (pSer19) of HSPB5 under ER stress conditions caused by proteome inhibitor MG132. This phosphorylation is responsible for inhibitory effect of HSPB5 on ER stress-induced apoptosis by MG132. PLK2/HSPB5 pathway provides a molecular mechanism for pSer19 of HSPB5 and represents part of mechanism for suppressing ER stress-induced apoptosis via proteasome inhibition.

In this study, we explored the binding proteins of HSPB5 and newly identified PLK2, which binds to HSPB5 under ER stress by MG132 treatment. PLK2 belongs to the polo-like kinase family and has recently been investigated as a therapeutic target for tumors [24]. We further showed that PLK2 phosphorylates serine 19, one of the three phosphorylation sites of HSPB5 [35,40], suppresses apoptosis induced by MG132-induced ER stress. Our findings provide new insights into cytoprotection by the PLK2/HSPB5 pathway in ER stress-induced cell apoptosis (Figure 8).

To explore the binding proteins of HSPB5, we employed BioID2 [20], a modified proximity-dependent biotin labeling method (Figure 2). Initially, we used a conventional method to purify biotinylated proteins with streptavidin beads [21]. However, conventional purification also isolated many non-biotinylated proteins under our conditions. To reduce the specific binding of proteins to streptavidin beads, we changed the purification method to the DiDBiT method [22], which purifies biotinylated peptides after trypsin digestion. This method suppressed specific binding to the beads by disrupting the protein’s three-dimensional structure. In addition, the low molecular weight peptides bind efficiently to the beads, reducing the volume of beads required to purify biotinylated peptides. By making these improvements in the purification step, we significantly improved the number of biotinylated peptides identified by mass spectrometry (Figure 2). The names of the proteins whose emPAI scores in mass spectrometry were increased by MG132 treatment are shown in the list (Table 1).

PLK2 is known to phosphorylate α-synuclein, one of the non-amyloid components that accumulate in neurological diseases such as Alzheimer’s [36]. PLK2 is also known to be regulated by CHOP [41], and we speculated that PLK2 might be involved in ER stress in muscle cells. The expression level of PLK2 was specifically upregulated with HSPB5 under ER stress after MG132 treatment in L6 cells (Figure 3b, Figure S2). The binding of PLK2 to HSPB5 in the ER region was validated (Figure 4c and Figure 5d). The labeling radius by BioID has been estimated to be 10 nm [20,21]. Therefore, proximity-dependent biotin labeling is considered to be affected by the orientation of the binding protein [13,42]. The biotinylation was concentrated in the kinase domain on the N-terminal side, indicating the region of PLK2 that binds to HSPB5 (Figure 4a). HSPB5 has three phosphorylation sites (serine 19, serine 45, and serine 59), but the kinase that phosphorylates serine 19 of HSPB5 is unknown [10,11]. This study confirmed that inhibition of PLK2 suppressed the ER stress-induced phosphorylation of serine 19 in HSPB5 (Figure 6).

PLK2 did not activate MK2 or p38 [40], which are known kinases that phosphorylate other serine residues of sHSP (Figure S5), and the MK2 inhibitor did not inhibit the phosphorylation of serine 19 induced by ER stress (Figure S6). These results confirm that PLK2 phosphorylates serine 19 of HSPB5 during ER stress. Although several substrates of PLK2 have been reported, the number of identified substrates is insufficient compared to other kinases; therefore, it is difficult to predict a consensus sequence of PLK2 substrates [43]. Further in vitro studies are needed to determine whether PLK2 is a kinase that directly phosphorylates pSer19 of HSPB5.

Inhibition of PLK2 also suppressed the MG132-induced translocation of HSPB5 from the cytoplasm to the ER region (Figure 4d). However, an amino acid substitution at serine 19 in HSPB5 did not suppress the translocation (Figure 7a). These results suggest that substrates other than HSPB5 phosphorylated by PLK2 may regulate the subcellular localization of HSPB5 via ER structure and vesicular trafficking. In the analysis of binding proteins by HSPB5-BioID (Table 1), the list of emPAI scores included ER transport proteins such as translocon-associated protein subunit beta (short name: TRAP-beta) and vesicle trafficking-related proteins such as dynactin subunit 2 (DCTN2), and ADP-ribosylation factor GTPase-activating protein 2 (ArfGAP2). These proteins may be phosphorylated by PLK2 under ER stress by MG132 treatment and affect the subcellular localization of HSPB5.

Desmin forms intermediate filaments and is involved in cellular force transmission and maintaining stable morphology of the muscle fibers. Protein degeneration due to abnormal aggregation of desmin has been implicated in such muscle disorders as atrophy and cardiomyopathy [29]. Desmin aggregates and HSPB5 are known to colocalize in pathological muscle tissues associated with desmin degeneration [44]. The knockdown of PLK2 suppressed the colocalization of HSPB5 with desmin aggregated by MG132 treatment (Figure 6b). This result is consistent with the previous studies showing that multiple phosphorylation is essential for the chaperone function of HSPB5 [45,46]. The detection of caspase 3 confirmed the cytoprotective effect of HSPB5 after the knockdown or overexpression of recombinant HSPB5 (Figure 7b,d). Furthermore, knockdown of PLK2 confirmed activation of PARP, which acts downstream of caspase 3 (Figure 7c).

HSPB5 (R120G) mutant, which causes desmin-related myopathy, presented no cytoprotective effect of HSPB5 [47]. In the comparison of HSPB5 mutants, the inhibitory effect of HSPB5 (wt) on apoptosis induced by MG132 treatment was inhibited by mutations in HSPB5 (3A) or HSPB5 (R120G). A single amino acid substitution in HSPB5 (S19A) also suggested an inhibitory effect similar to HSPB5 (3A) (Figure S7). These results suggest that phosphorylation of HSPB5, including pSer19 by PLK2, contributes to the stability of desmin against ER stress in muscle cells. This phosphorylation is expected to have some cytoprotection against ER stress caused by proteasome inhibition.

## 4. Materials and Methods

### 4.1. Reagents

The following antibodies were used for immunostaining or immunofluorescence applications: HSPB5 (NBP1-47708, Novus Biologicals, Centennial, CO, USA), pHSPB5 (pSer19; C7740, Sigma-Aldrich Japan KK, Tokyo, Japan), pHSPB5 (pSer45; NB120-5598, Novus), pHSPB5 (pSer59; ab5577, Abcam, KK, Tokyo, Japan), β-tubulin (014-25041, Fujifilm Wako Pure Chemicals, Osaka, Japan), p38 (612168, BD Transduction Laboratories, Franklin Lakes CO, USA), and pp38 (pThr180/pTyr182; 612280, BD). PLK2 (14812), CHOP (2895), PDI (3501), MK2 (3042), pMK2(pThe334; 3041), caspase 3 (14220), cleaved-caspase3 (9664), PARP (9542), cleaved-PARP (94885), desmin (5332) were purchased from Cell Signaling Technology (Danvers, MA, USA). Antibodies against tag sequences were FLAG (1E6, Fujifilm Wako) and HA (for Western blotting; #4B2, Fujifilm Wako, for tissue immunostaining; 3F10, Sigma-Aldrich). The secondary antibodies used were horseradish peroxidase (HRP)-conjugated antibody (Jackson Immunoresearch Laboratories, West Grove, PA, USA) for Western blotting and Alexa Fluor 488 or 546-conjugated antibody (Thermo Fisher Scientific KK, Tokyo, Japan) for fluorescent immunostaining. The following pharmacological reagents were used: MG132 (CS-0471, Chemscene LLC, South Brunswick Township, NJ, USA), PLK inhibitor BI2536 (CS-0071, Chemscene LLC), MK2 inhibitor III (15943, Cayman Chemical, Ann Arbor, MI, USA), tunicamycin (11445, Cayman), streptavidin-peroxidase polymer (S2438, Sigma-Aldrich), streptavidin-Alexa Fluor 546 (S11225, Thermo Fisher Scientific), and DAPI (Nacalai Tesque, Kyoto, Japan). siRNA duplexes for rat HSPB5 cDNA (SASI_Mm01_00130172 and SASI_Mm01_00130177) and MISSION siRNA Universal Negative Control 1 were purchased from Sigma-Aldrich. siRNA duplexes for rat PLK2 cDNA (Silencer select 4390771) and negative control 1 were purchased from Thermo Fisher Scientific.

### 4.2. Plasmid Construction

cDNA of HSPB5 (NCBI No. NM_001289782) were amplified using a KOD One PCR kit (Toyobo, Osaka, Japan) and subcloned into the *Bam*H I site of the p3×FLAG CMV-10 (Sigma-Aldrich) or MCS-BioID2-HA [14] (Addgene plasmid 74224) using in-fusion systems (Takara Bio, Shiga, Japan). Mutant HSPB5 (S19A), HSPB5 (S45A), HSPB5 (S59A), HSPB5 (3A; S19A, S45A, and S59A), and HSPB5 (R120G) [12] were also prepared by PCR based on in-fusion cloning.

### 4.3. Cell Culture

L6, 293T, and HeLa cells were cultured in DMEM (044-29765, Fujifilm Wako) supplemented with 10% fetal bovine serum medium, 100 IU/mL penicillin, and 100 μg/mL streptomycin (Fujifilm Wako). Muscular differentiation of L6 cells was induced by culturing the cells in DMEM supplemented with 2% horse serum (Thermo Fisher Scientific) for 48 h [48]. ER stress was induced by the medium exchange in a culture medium containing 5 µM MG132. According to the manufacturer’s instructions, plasmids and siRNA were transfected into cells using Lipofectamine 3000 or Lipofectamine RNAiMAX (Thermo Fisher Scientific). Cell viability and cytotoxicity rates were measured using Cell Counting Kit-8 (Fujifilm Wako) and Cytotoxicity LDH Assay Kit-WST (Fujifilm Wako) according to the manufacturer’s instructions.

### 4.4. Identification of Binding Proteins Using the BioID Method

Cells overexpressing BioID2 fusion protein were solubilized with RIPA buffer (25 mM Tris-HCl pH 7.5, 150 mM NaCl, 1% Nonidet-P40, 1% sodium deoxycholate, 0.1% SDS) containing protease inhibitor cocktails and phosphatase inhibitor cocktails (Fujifilm Wako).and incubated for 20 min at −20 °C with four times the volume of cold acetone, followed by centrifugation (15,000 rpm, 10 min at 4 °C) to obtain acetone precipitates. The pellets were carefully washed with 75% acetone, thoroughly dried, dissolved in 4 M urea, 50 mM NH_4_HCO_3_, and 10 mM DTT solution, and incubated at 55 °C with vigorous orbital shaking for 30 min. Protein alkylation was performed by adding 20 mM iodoacetamide and incubating for 30 min in the dark. To digest the biotinylated proteins, we added 0.02% ProteaseMAX (Promega Japan, Tokyo, Japan) and porcine trypsin protease MS grade (90057; Thermo Fisher Scientific) to the solution (enzyme/protein is 1/100) and incubated at 37 °C for 3 h. Digestion was stopped by adding trifluoroacetic acid (TFA) to the reaction solution to a final concentration of 0.1%. Peptides were desalted using an SPE C18 Hypersep column (bed weight, 50 mg; column volume, 1 mL; Thermo Fisher Scientific), and each 50% acetonitrile eluent was collected into a TPX tube (IEDA Trading, Tokyo, Japan) and dried in a vacuum centrifuge. Peptides were dissolved in lysis buffer (20 mM Tris-HCl pH 7.4, 0.5% Nonidet-P40, 200 mM NaCl, 2.5 mM MgCl_2_), suspended in tamavidin 2-REV magnetic beads (136-18341; Fujifilm Wako), rotated at 4 °C for 4 h, and then the beads were washed three times with lysis buffer. The beads were transferred to TPX tubes, washed with distilled water, and incubated with 80% acetonitrile/0.1% TFA at room temperature for 3 min. As previously described, the collected biotinylated peptides were subjected to nanoLC/MS/MS [49]. MS/MS data files were interpreted using MASCOT (Ver. 2.3.02) MS/MS ion searches (Matrix Science, London, UK) against 20,317 sequences of human proteins in the SwissProt 2018_03 database. The peptide and fragment mass tolerances were set to 4 ppm and 0.8 Da, respectively. Fixed and variable modifications were set as carbamidomethyl of cysteine residue and biotinylation of a lysine residue, respectively.

### 4.5. Western Blot Analysis

Tissues or cells were lysed by sonication (15 s, four times) with an ultrasonic disrupter in RIPA buffer containing protease inhibitor cocktails and phosphatase inhibitor cocktails (Fujifilm Wako). Equal amounts of protein were subjected to SDS-polyacrylamide gel electrophoresis and electro-transferred to a PVDF membrane (Immobilon-P; pore size 0.45 µm, Merck KK, Tokyo, Japan). The bound antibodies on the membrane were detected by chemiluminescence with horseradish peroxidase (HRP)-conjugated secondary antibody (Jackson Immunoresearch Laboratories, PA, USA) and ImmunoStar Zeta detection reagents (Fujifilm Wako) on the Limited-STAGE (AMZ system science, Osaka, Japan). Applied protein were confirmed by immunoblotting with β-tubulin antibody, as described previously [50].

### 4.6. HSPB5 Binding Assay

L6 cells overexpressing FLGA-HSPB5 were quickly washed with ice-cold PBS (−), solubilized with lysis buffer for the binding assay (50 mM Tris-HCl pH 7.5, 2 mM MgCl_2_, 150 mM NaCl, 0.5% Nonidet-P40, 1 mM EDTA, 1 mM Na_2_VO_4_, and 1 mM NaF), and the supernatant was collected after centrifugation (16,000× g, 10 min, 4 °C). The supernatant was immunoprecipitated with anti-tag antibody-conjugated beads (012-22781, Fujifilm Wako), washed three times with lysis buffer for the binding assay, and the samples were subjected to Western blotting.

### 4.7. Immunofluorescence Microscopy

L6 cells cultured in a Cellview glass-bottom dish (#627860, Greiner Japan, Tokyo, Japan) were fixed with 10% formaldehyde/PBS (−) for 10 min, permeabilized with 0.1% Triton X-100/PBS (−) for 5 min, washed with phosphate-buffered saline with Tween 20, and incubated with each primary antibody for 1 h. Fluorescence staining was performed using Alexa Fluor dye conjugated secondary antibodies (Thermo Fisher Scientific) and DAPI solution. Images were acquired using a confocal laser-scanning microscope with Zeiss Zen imaging software (LSM700; Zeiss Japan, Tokyo, Japan) [51]. Immunofluorescence staining with BioID was performed as described previously [15].

### 4.8. Statistical Analyses

The density of protein bands detected by Western blotting was measured using ImageJ (National Institutes of Health, Bethesda, MD, USA). For overexpression, siRNA, or cell viability assays, biological replicates were performed using three different samples. Three technical replicates were performed for the pull-down assay of HPSB5 and PLK2. Statistical significance was determined at *p* < 0.05 on Student’s *t*-test using Excel 2019 (Microsoft Japan, Tokyo, Japan). Multiple comparisons were performed using the Tukey method with BellCurve (Social Survey Research Information, Tokyo, Japan) [52].

## 5. Conclusions

In this study, we applied the BioID method to analyze the binding proteins of HSPB5 under ER stress via proteasome inhibition by MG132 treatment. We found that PLK2, a member of the polo-like kinase family, binds to HSPB5. Inhibition of PLK2 kinase activity suppresses the phosphorylation of serine 19 of HSPB5 by MG132 treatment and prevents translocation of HSPB5 to the ER region. pSer19 is not required for the translocation of HSPB5, suggesting that another phosphorylated substrate of PLK2 is involved in the subcellular localization of HSPB5 after MG132 treatment. HSPB5 and PLK2 contribute to suppressing caspase 3/PAPR activation in the apoptosis by MG132 treatment, and pSer19 of HSPB5 is involved in this mechanism. This study suggests that PLK2 is essential in cytoprotection against MG132-induced ER stress via pSer19 of HSPB5.

## Figures and Tables

**Table 1:** List of candidate binding proteins for HSPB5 identified by DiDBit method.

	emPAI ^a^	
Protein Name	MG132 (−)	MG132 (+)
PHD finger-like domain-containing protein 5A	0	0.26
Poly(rC)-binding protein 2	0	0.18
Translocon-associated protein subunit beta	0	0.17
Dynactin subunit 2	0	0.15
Serine/threonine-protein kinase PLK2	0	0.13
6-phosphogluconate dehydrogenase,	0	0.13
ADP-ribosylation factor GTPase-activating protein 2	0	0.12
Pyruvate kinase PKM	0.06	0.18
Paired mesoderm homeobox protein 1	0	0.12
Eukaryotic initiation factor 4A-I	0.07	0.15
Phosphoglycerate kinase 1	0	0.07
26S proteasome regulatory subunit 6A	0	0.07
Sequestosome-1	0.07	0.14
Abl interactor 1	0	0.06
PDZ and LIM domain protein 7	0	0.06
Heat shock cognate 71 kDa protein	0	0.05

^a^ emPAI is an exponential index of the absolute concentration of protein from the number of peptides detected by mass spectrometry.

## Data Availability

The data presented in this study are available in the article.

## Supplementary Materials

The supporting information can be downloaded at: https://www.mdpi.com/article/10.3390/ijms231911257/s1.

## Abbreviations

emPAI	exponentially modified protein abundance index
PLK2	polo-like kinase 2
CHOP	C/EBP homologous protein
siRNA	small interference RNA
BioID	proximity-dependent biotin identification
nanoLC/MS/MS	nanoscale liquid chromatography coupled to tandem mass spectrometry
MK2	MAP kinase-activated protein kinase 2
p38	p38 MAP kinases
siRNA	small interference RNA
TFA	trifluoroacetic acid
DAPI	6-diamidino-2-phenylindole
PARP	poly ADP-ribose polymerase
