# Supplementary material for: Polo-Like Kinase 2 Plays an Essential Role in Cytoprotection against MG132-Induced Proteasome Inhibition via Phosphorylation of Serine 19 in HSPB5
Source: Int J Mol Sci. 2022 Sep 24;23(19):11257. doi: 10.3390/ijms231911257 (PMC9569985; doi:10.3390/ijms231911257)

Supplementary data

Figure S1: Peptide sequence of biotinylated lysine of PLK2 detected by mass spectrometry.

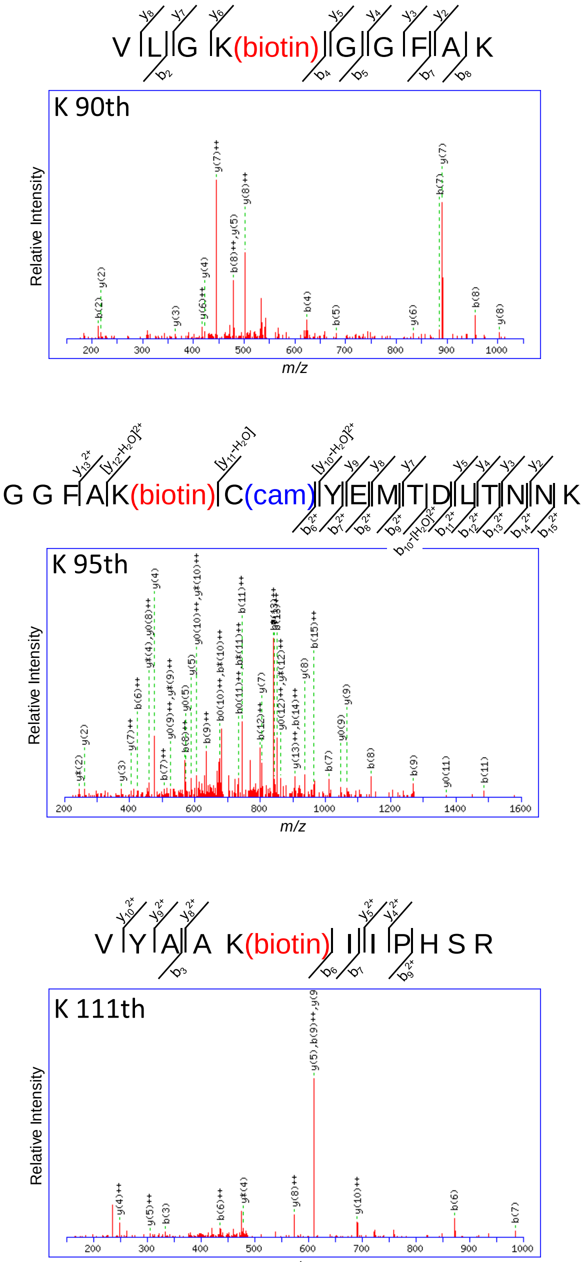

PLK2 (NCBI No. NP_006613)

```
MELLRTITYQ PAASTKMCEQ ALGKGCGADS KKKRPPQPPE ESQPPQSQAQ VPPAAPHHHH  60
HHSHSGPEIS RIIVDPTTGK RYCRGKVLGK GGFAKCYEMT DLTNNKVYAA KIIPHSRVAK 120
PHQREKIDKE IELHRILHHK HVVQFYHYFE DKENIYILLE YCSRRSMAHI LKARKVLTEP 180
EVRYYLRQIV SGLKYLHEQE ILHRDLKLGN FFINEAMELK VGDFGLAARL EPLEHRRRTI 240
CGTPNYLSPE VLNKQGHGCE SDIWALGCVM YTMLLGRPPF ETTNLKETYR CIREARYTMP 300
SSLLAPAKHL IASMLSKNPE DRPSLDDIIR HDFFLQGFTP DRLSSSCCHT VPDFHLSSPA 360
KNFFKKAAAA LFGGKKDKAR YIDTHNRVSK EDEDIYKLRH DLKKTSITQQ PSKHRTDEEL 420
QPPTTTVARS GTPAVENKQQ IGDAIRMIVR GTLGSCSSSS ECLEDSTMGS VADTVARVLR 480
GCLENMPEAD CIPKEQLSTS FQWVTKWVDY SNKYGFGYQL SDHTVGVLFN NGAHMSLLPD 540
KKTVHYYAEL GQCSVFPATD APEQFISQVT VLKYFSHYME ENLMDGGDLP SVTDIRRPRL 600
YLLQWLKSDK ALMMLFNDGT FQVNFYHDHT KIIICSQNEE YLLTYINEDR ISTTFRLTTL 660
LMSGCSSELK NRMEYALNML LQRCN
```

Figure S2: Induction of PLK2 and HSPB5 expression under ER stress by MG132 treatment. Undifferentiated L6 cells were treated with 5 μM MG132. The induction of PLK2, HSPB5 and GAPDH protein expression was detected by western blotting.

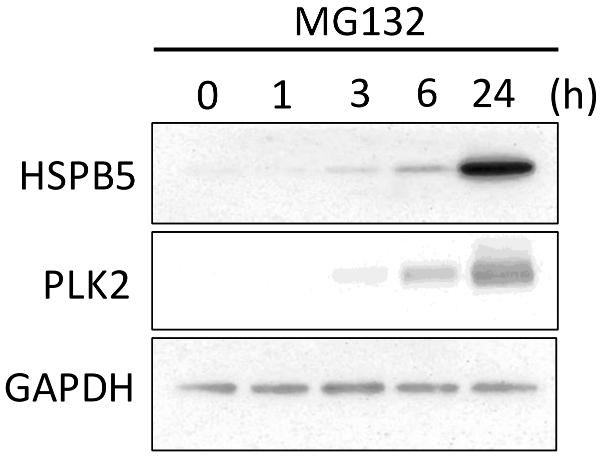

Figure S3: Induction of HSPB5 and PLK2 expression under ER stress by bortezomib treatment. Undifferentiated L6 cells were treated with 5 μM MG132 or 1 μM bortezomib (#A10160, Adooq Bioscience, CA, USA). The induction of HSPB5 and PLK2 protein expression was detected by western blotting. The graph shows the expression level of endogenous HSPB5 and PLK2. Data represent the mean ± standard error (n = 3). Statistical analyses were performed using One-way ANOVA with Tukey test. ‡p < 0.01.

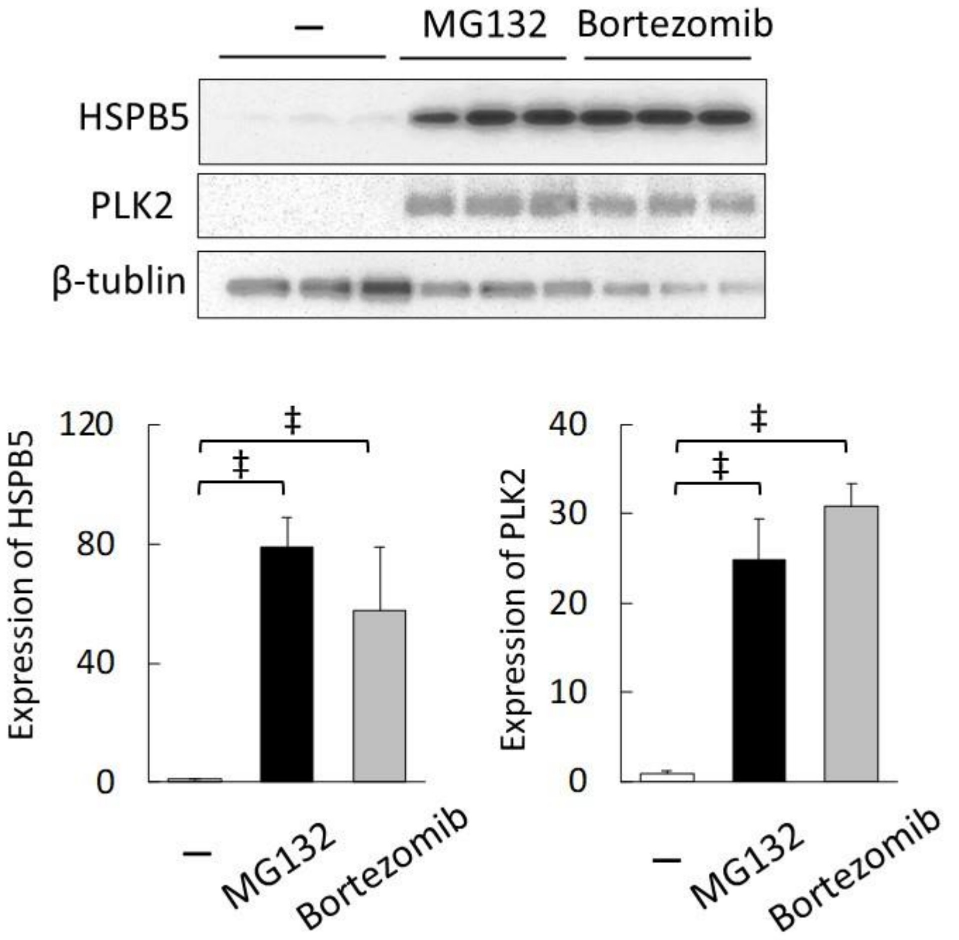

Figure S4: HSPB5 co-localizes with the ER marker, protein disulfide isomerase (PDI), by MG132 treatment. After 5 μM MG132 treatment (24 h), FLAG-tagged HSPB5 and endogenous PDI in undifferentiated L6 cells were immunofluorescently stained with anti- tag (#1E6, Fujifilm Wako) and anti-PDI (#3501, Cell Signaling Technology) antibodies. Nuclei were fluorescently stained with DAPI solution. Yellow color in Merge image indicates co-localization of HSPB5(WT) and PDI.

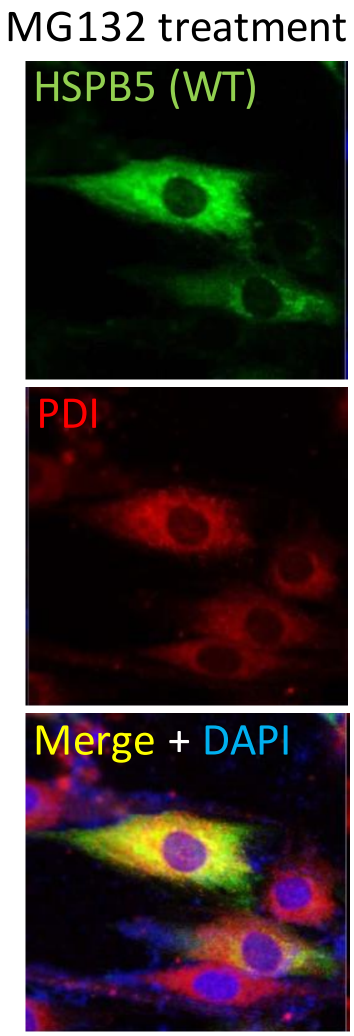

Figure S5. PLK2 is not involved in the activation of p38 and MK2.

After MG132 treatment (24 h), phosphorylation of MK2 (pThe334) and p38 (pThr180/pTyr182) was detected by western blotting. The graph shows the phosphorylation level of endogenous MK2 and p38. Data represent the mean ± standard error (n = 3). Statistical analyses were performed using One-way ANOVA with *t*-test. n.s. means not significant.

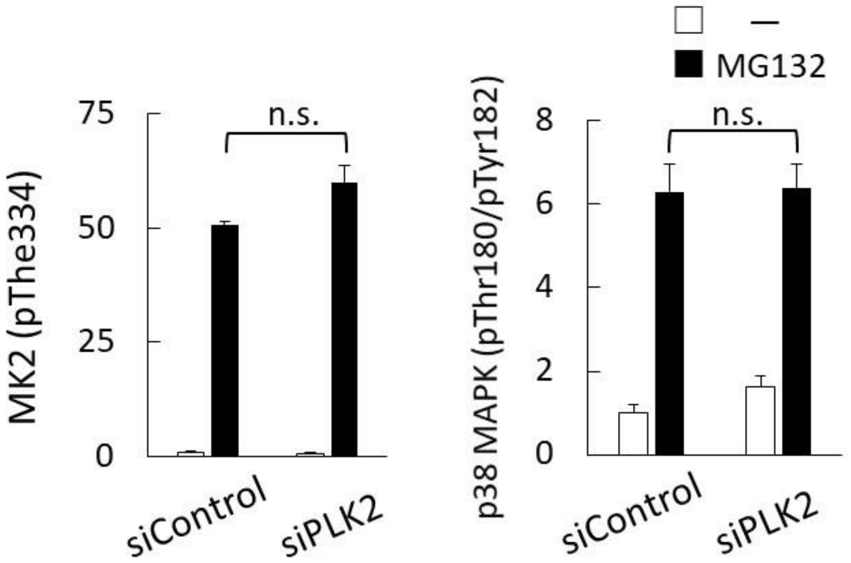

Figure S6: MK2 does not phosphorylate serine 19 of HSPB5.

After 5 μM MG132 treatment (24 h) with various concentrations of MK2 inhibitors, phosphorylation of HSPB5 (pSer19) was detected by western blotting.

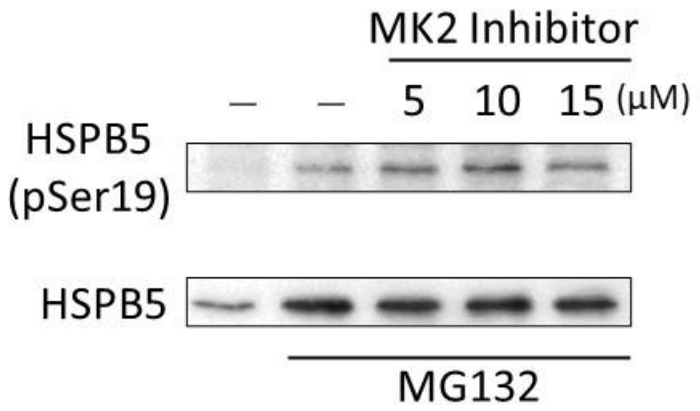

Figure S7: Comparison of the inhibitory effect of HSPB5 phosphorylation on ER stress-induced caspase-3 activity.

(a) Detection of cleaved caspase-3 positive cells in L6 cells expressing recombinant protein. Undifferentiated L6 cells were transfected with expression plasmids by lipofection. HSPB5 (3A) is a recombinant protein with triple point mutations in S19A, S45A, and S59A. Control cells were transfected with empty expression vectors. After 48 h of lipofection, undifferentiated L6 cells were treated with 5 μM MG132 for 16 h. FLAG-tagged HSPB5 were immunofluorescently stained with anti-tag antibodies. Caspase-3 activity was detected using a fluorescently labeled antibody specific for cleaved caspase-3 Asp175 (#9661, Cell Signaling Technology). Fluorescent stained images of cells were observed using a confocal laser scanning microscope (LSM700, Zeiss Japan). Arrows in the column of cleaved caspase-3 indicate cells expressing recombinant HSPB5. White-framed arrows indicate cleaved caspase-3 positive cells. Blue arrows indicate non-positive cells. Scale bar indicates 20 μm. **(b)** The graph shows the percentage of cleaved caspase-3 positive cells divided by the total number of cells (control) or the percentage of cleaved caspase-3 positive cells expressing HSPB5 divided by the total number of HSPB5 expressing cells (HSPB5). The mean of three independent experiments is shown. Statistical analyses were performed using One-way ANOVA with Tukey test. ‡$p < 0.01$; n.s. means not significant.

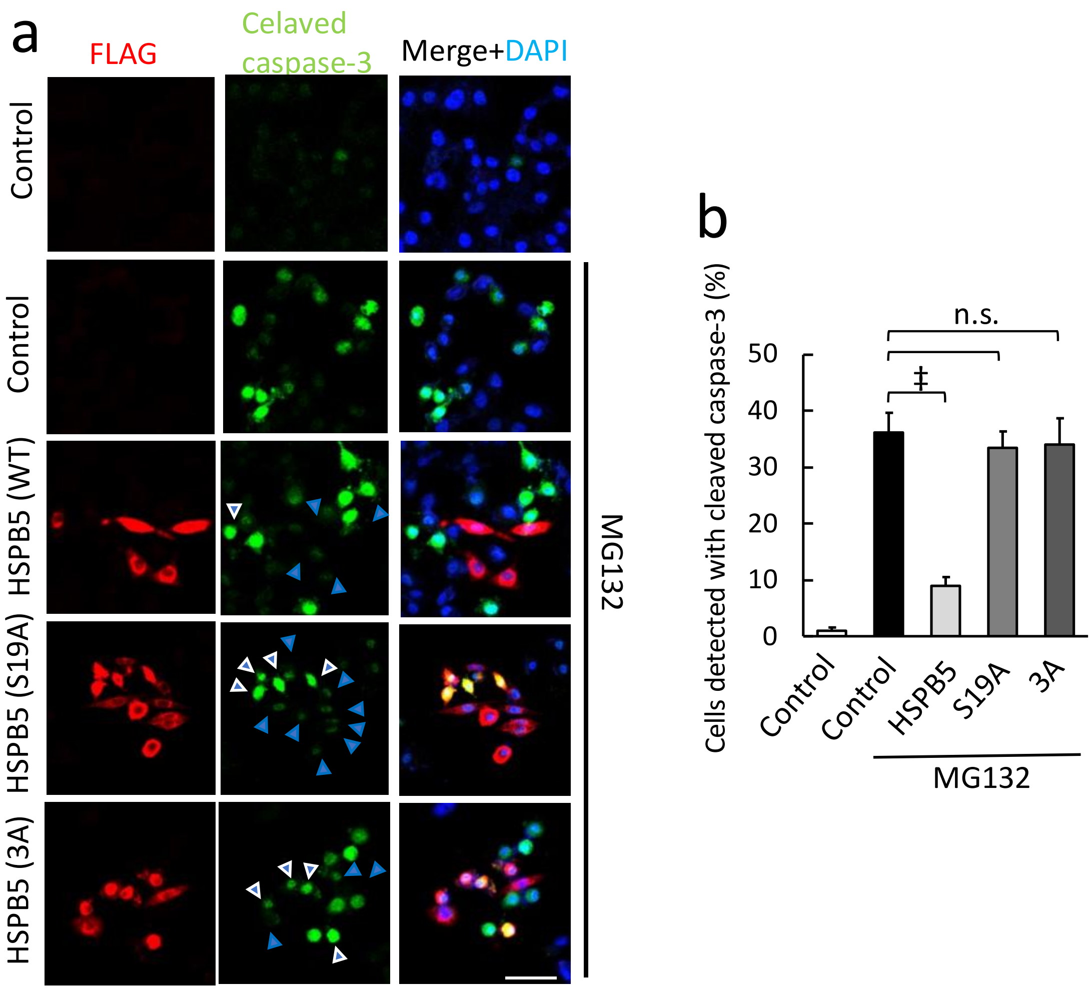

Supplement: Supplementary file 1 [file ijms-23-11257-s001.zip › ijms-1359308-supplementary.pdf]
